# Perception, Transduction and Crosstalk of Auxin and Cytokinin Signals

**DOI:** 10.3390/ijms232113150

**Published:** 2022-10-29

**Authors:** Georgy A. Romanov

**Affiliations:** Timiryazev Institute of Plant Physiology, Russian Academy of Sciences, 127276 Moscow, Russia; gar@ippras.ru or gromanov@yahoo.com

Auxins and cytokinins are considered the most important plant hormones, responsible for fundamental traits of the plant organism [1]. These hormones determine the uniqueness of the plant hormonal system: the main sites of their synthesis are at opposite poles of the plant body; auxins are synthesized at the top, and cytokinins, at the bottom. From their sites of synthesis, they move along the main plant axis in opposite directions. This pivotal auxin–cytokinin countercurrent creates hormonal gradients that affect cell behavior throughout the plant’s life. Both hormones act synergistically in stimulating cell division, but antagonistically in shoot or root branching. Thus, these two hormones largely determine the plant’s phenotype. Other important plant traits, including resistance to biotic and abiotic stresses, are also auxin-/cytokinin-dependent. The 21st century has seen great progress in dissecting the molecular mechanisms of auxin/cytokinin perception and signal transduction. Both mechanisms have been shown to be specific for plants; they do not mirror the mechanisms described for animal hormones. However, many important details of auxin and cytokinin signaling and interplay remain obscure, attracting great interest from researchers. Some of these challenges have been addressed by the authors of the *IJMS* Special Issue “Perception, Transduction and Crosstalk of Auxin and Cytokinin Signals” (2021–2022).

**Reconstitution of the Cytokinin Signaling Pathway**. Cytokinin signaling proteins are encoded by multigene families; therefore, determining the contribution of each component to a signaling pathway is challenging. This was addressed by researchers from the National Institute of Agricultural Sciences (Jeonju, Korea), who succeeded in reconstituting the cytokinin signaling pathway by the transient expression of genes encoding major players in the signal transduction chain in rice protoplasts [2]. Thereby, the roles of the receptor (OsHK03), phosphotransmitters (OsHP01-03 and OsHP05) and type-B response regulators (OsRR16-19) were quantitatively assessed using a luciferase reporter gene construct driven by synthetic cytokinin-responsive promoter TCSn [3]. This new system allows the exploration of various combinations of major cytokinin signaling components in living rice cells in a single experiment. In light of the proven positive effects of cytokinin on seed yield [4], fine-tuning cytokinin signaling by selecting the best combination of signaling genes controlled by optimized promoters may be promising for further improving rice productivity.

**Evolution of the Cytokinin Perception Apparatus**. Although the cytokinin signaling in typical model plants, such as Arabidopsis or rice, has been studied in-depth, the investigation of its evolution remains in its infancy. Hormone perception is based on sensing receptor proteins, so receptors are key components of signaling cascades. Lomin et al. (2021) [5], from the Timiryazev Institute of Plant Physiology (Moscow, Russia), with the participation of Dr. Heyl (Adelphi University, Garden City, USA), set out to investigate the cytokinin perception apparatus in early divergent plant species such as the bryophyte *Physcomitrium patens*, lycophyte *Selaginella moellendorffii*, and gymnosperm *Picea abies*. All the receptors or their individual sensory modules were analyzed in terms of their cytokinin-binding affinities, their ligand specificity, and the pH-dependencies of hormone binding. Through this integrative approach, new trends in the evolution of land-plant cytokinin receptors were identified. In particular, moss receptors were shown to strongly prefer isopentenyladenine (iP) over other forms of cytokinin nucleobases, whereas spruce receptors bound *trans*-zeatin (tZ) with much higher affinity than iP. These trends are consistent with the parallel evolution of active cytokinin forms which, in primitive plants, are mainly represented by iP, whereas in vascular plants starting from gymnosperms, specific enzymes (CYP735-like) for the direct synthesis of tZ from iP-type precursors appear.

**How Do Cytokinins Get to Their Receptors?** One of the recurring problems in cytokinin signaling research is the subcellular distribution of cytokinins between different cellular compartments. It is fundamentally important for any hormone, such as a cytokinin, to have access to a cognate receptor. The main cellular targets of cytokinins are currently thought to be the lumens of the endoplasmic reticulum (ER), where the sensory modules of the receptors are exposed [6,7]. For optimal plant growth and development, it is necessary for a sufficient amount of hormone to reach the receptor at the right time. A research team from the Institute of the Experimental Botany, Prague [8], presents a broad panorama of internal and external factors that can determine the location of each particular version of a cytokinin in the cell. The review provides much unique basic information on the physicochemical properties of cytokinins, data on their subcellular localization and a detailed survey on cytokinin transporters, and highlights the problems remaining to be solved. 

**Environmental Cues Modulate Cytokinin Signaling**. Plants’ survival in different climatic zones requires effective adaptation. The restructuring of the cytokinin regulatory system can play an important role in this [9]. A team from the Czech Republic (with a participant from Hungary) led by researchers from the Prague Institute of Experimental Botany studied the effect of light quality and intensity on cold acclimation in Arabidopsis [10]. They found that among the known phytohormones, cytokinins appeared to be principal players. The results showed that cold acclimation at optimal light intensity was associated with the upregulation of tZ signaling in leaves and roots, while a combination of cold and low light was associated with an increase in *cis*-zeatin signaling in apices. Since the publication of the article [10], this scientific field has experienced further rapid progress. 

**Synthetic Drugs with Unique Cytokinin or Anticytokinin Properties**. The development of specialized methods for the analysis of cytokinin–receptor interactions [11,12] created the preconditions for the generation of cytokinins with improved properties, prospective for use in practical crop production. One of the directions of such work was the creation of artificial cytokinins specific to only one particular version of the receptor isoforms. This task proved to be challenging and was only recently completed by the joint efforts of researchers from two Moscow Institutes: biologists from the Institute of Plant Physiology and chemists from the Institute of Molecular Biology. In this work [13], the interaction of chiral derivatives of the aromatic cytokinin *N*6-benzyladenine with individual Arabidopsis receptors was studied. Three synthetic cytokinin *S*-enantiomers specific for AHK3 receptors were discovered: *N*6-((*S*)-α-methylbenzyl)adenine (*S*-MBA), 2-fluoro-*S*-MBA (*S*-FMBA) and 2-chloro-*S*-MBA (*S*-CMBA). Two other *S*-enantiomers were found to exhibit receptor-specific and chirality-dependent anticytokinin properties. These anticytokinins appear to act as allosteric regulators of the receptor activity. 

**Cytokinins + Auxins: Synergistic Morphogenic Effects.** One of the most striking effects of cytokinins, observed almost immediately after the discovery of these hormones in 1955, is the ability, together with auxins, to induce organogenesis in undifferentiated callus tissue [14]. A high auxin/cytokinin ratio promoted the emergence of roots, while a low ratio promoted the emergence of shoots. The morphogenic role of the key duo, cytokinin/auxin, in the course of shoot formation was the subject of a comprehensive review by a multinational team of authors from Serbia, the Czech Republic and China, led by Dr. Raspor from the University of Belgrade, Serbia [15]. The uniqueness of this review lies in the integrative approach to its creation. The authors consider each environmental cue in terms of its influence on hormonal signaling and on the expression of genes necessary for shoot regeneration. Processes that are usually overlooked, such as the uptake of hormones from the environment and their intercellular transport, are highlighted. Another aspect noted is the role of sucrose, which is usually added to the regeneration medium as a source of energy and carbon. The review shows that sucrose plays an active role in the processes of organogenesis, affecting hormone signaling, the spectrum of genes with altered expression, and probably also the uptake of hormones by cells. Thus, the authors of the review consider the whole complex of processes and interactions at the molecular level, believing that only such an integrative approach can provide an exhaustive explanation of the hormonal induction of de novo shoot formation. 

**Cytokinins + Auxins: Antagonistic Growth Effects.** Despite the true synergistic effects in stimulating cell proliferation, auxin and cytokinin most often act as an antagonistic hormone pair [1]. The critical role of this antagonism for plant growth and development under optimal and stressful conditions was well demonstrated in a review by Kurepa and Smalle from the University of Kentucky, (Lexington, USA) [16]. As a typical example of auxin/cytokinin antagonism, the authors chose the regulation of the shoot/root growth ratio, in which cytokinin promotes shoot and inhibits root growth, whereas auxin does the opposite. Recall that control of the shoot/root growth ratio is essential for the survival of land plants. This follows from the fact that a decrease in shoot growth combined with an increase in root growth contributes to survival under conditions of drought and nutrient deficiency. Consequently, it can be inferred that auxin promotes and cytokinin reduces drought tolerance and nutrient uptake. In addition, the review demonstrates recent data on the impacts of drought stress and nutrient availability on the cytokinin and auxin signaling and biosynthetic pathways. It was concluded that each of the two hormones directly and negatively regulates the biosynthesis and/or signaling of the other, with auxin playing a dominant regulatory role in this hormonal pair. 

**Cytokinins + Auxins: Adaptation to Photoperiod Stress**. Another example of a stress effect under the influence of the auxin–cytokinin duo was described in an article by a joint German–Czech group headed by Prof. Schmülling from the Free University of Berlin (Germany) [17]. In this case, we refer to the stress caused by light, more precisely, by an altered photoperiod due to the elongation of the light period. Arabidopsis plants were subjected to photoperiod stress using mutants for genes of the cytokinin or auxin regulatory systems along with wild-type plants. The stress level was determined by analyzing changes in gene expression patterns. It was found that *ahk2,3* mutants with loss of cytokinin receptor function, or gain-of-function *YUC1* auxin synthesis mutants exhibited increased stress responses, while mutations causing the loss of the function of the auxin receptors TIR1, AFB2 and AFB3, in contrast, reduced stress levels. Thus, cytokinin signaling has been shown to play a protective role in this particular type of light stress, whereas auxin signaling enhanced the stress effect. This finding was in good agreement with earlier data on transgenic *PHYB* potatoes, which showed that cytokinin (kinetin) could reduce plant sensitivity to photoperiod [18]. 

**Cytokinins + Auxins: Signaling Crosstalk at the Molecular Level.** In the aforementioned research on the concerted action of cytokinins and auxins, the main topics were somehow indirectly related to the interplay between auxin and cytokinin signaling. Unlike these studies, the main purpose of the work by Kolachevskaya et al. [19] from the Moscow Institute of Plant Physiology was a direct study of this crosstalk. The study was performed on potato plants grown in vitro on a medium with a low (vegetative growth stage) or high (tuber formation stage) sucrose concentration. The plants were exposed to moderate concentrations (1 µM) of auxin or cytokinin for 1 h, and then, the expression levels of genes involved in cytokinin or auxin signaling were determined. The results showed that auxins and cytokinins reciprocally influenced the signaling outputs of the respective pathways. These hormonal effects were mainly organ-specific and largely dependent on the sucrose content. Auxin acted as a negative regulator of cytokinin perception at all concentrations of sucrose in the media. By contrast, cytokinins antagonized auxin signaling only under low sucrose, whereas under high sucrose, they acted differently. The gathered data [19] point to functioning of multiple molecular links between cytokinin and auxin signaling modules.

**MicroRNA Fine-Tunes Auxin Signaling.** miRNAs are short, single-stranded nucleic acid molecules that repress target gene expression, regulating plant growth and stress responses [20]. The genes involved in auxin signaling are targeted by multiple miRNAs, and some of these genes are directly involved in growth and stress reactions. In the informative overview by a Chinese team mainly from Gansu Agricultural University and the Institute of Soil Sciences [21], the miRNA-mediated regulation of auxin signaling is divided into two types: (i) the direct targeting of the genes involved in the auxin signaling pathway, including *TIR1/AFBs*, *ARF* and *AUX/IAA*; (ii) the modulation of the free auxin content via the indirect regulation of auxin biosynthesis, metabolism and transport. The authors consider the latest studies on the miRNA-mediated regulation of the auxin pathway in the dicot Arabidopsis and the monocot rice, as well as new findings in other species, in order to provide a comprehensive picture of the important roles that miRNAs play in auxin signaling. The combination of certain miRNAs with the genes involved in auxin signaling creates specific regulatory modules that fine-tune development and stress responses in the plant kingdom. Some of these modules function as potent regulatory nodes to integrate auxin, cytokinin and other signaling pathways. Such regulation appears in a wide range of developmental processes in plants. The challenges remaining to be addressed are highlighted. 

**Concluding remarks.** This Special Issue, “Perception, Transduction and Crosstalk of Auxin and Cytokinin Signals”, was aimed at bringing together researchers studying various aspects of the molecular basis of auxin and/or cytokinin perception and signal transduction (Figure 1). Indeed, scientists from China, the USA, the Czech Republic, Russia, Germany, Korea, Serbia and Hungary took part in this Special Issue with their valuable contributions. The research on this topic is quickly developing, and this Special Issue will certainly attract the attention of plant biologists due to the central role that auxin and cytokinin play in plant growth, development and stress resistance. This Special Issue will serve to promote such studies and make them more visible to the scientific community.

## Figures and Tables

**Figure 1:**
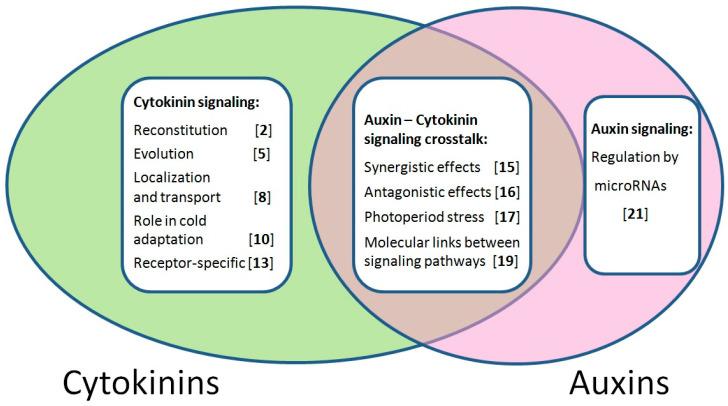
Venn diagram summarizing the *IJMS* Special Issue “Perception, Transduction and Crosstalk of Auxin and Cytokinin Signals” [2,5,8,10,13,15,16,17,19,21]. Areas associated with cytokinin and auxin are marked in green and pink, respectively; the overlapping region corresponds to both hormones. Numbers on white desks correspond to the order of the references in this Editorial. Captions serve as indicators of the contents of the articles included in the Special Issue.
